# A novel bladder cancer surveillance schedule using bladder Cx for patients on annual surveillance

**DOI:** 10.1002/bco2.468

**Published:** 2025-01-15

**Authors:** Arjun Guduguntla, Thomas Whish‐Wilson, Lauren Chandler, Dennis Gyomber

**Affiliations:** ^1^ Department of Urology Northern Health Victoria Australia; ^2^ University of Melbourne Victoria Australia

**Keywords:** bladder cancer surveillance, bladder cx, bladder cx monitor, non‐muscle invasive bladder cancer, skip cystoscopy, urine test

## Abstract

**Objectives:**

Cx bladder monitor (CxM) is a urine test with a proven high sensitivity and negative predictive value in bladder cancer surveillance. The aim of this retrospective study was to report on the outcomes of our newly implemented bladder cancer surveillance program for patients eligible for yearly cystoscopy, as per the European Association of Urology (EAU) guidelines. In this program, eligible patients alternate between yearly surveillance cystoscopy and CxM, instead of the standard yearly surveillance cystoscopy. Outcomes measures were overall results of CxM and subsequent treatment patterns of patients, impact on waiting lists, cost comparison and patient satisfaction.

**Patients and Methods:**

In 2022, 109 eligible and consenting patients were identified, with 98 commencing on the new surveillance program, starting with CxM instead of cystoscopy. A negative CxM, would result in a planned flexible cystoscopy in 12 months. If a patient had a positive CxM, they proceeded to undergo a cystoscopy, and if required, imaging.

**Results:**

Of the 98 that underwent testing, 90 had a negative CxM test and 8 patients had a positive CxM test. Three of these eight were true positive (PPV 0.375). Seventy negative CxM patients had no recurrence at the time of the next cystoscopy/imaging. Of the remaining 20 negative CxM patients, 11 were found to have a recurrence at subsequent cystoscopy/imaging and 9 did not proceed with further surveillance for various reasons. All of the tumour recurrences diagnosed after a negative CxM were non‐invasive, thus there was no progression to muscle‐invasive disease. All suitable patients consented to continuing with the CxM protocol. The hospital surveillance cystoscopy waitlist was reduced by approximately 59% and CxM was approximately $850 AUD cheaper than a cystoscopy.

**Conclusion:**

CxM can be safely used in an alternating schedule with Flexible Cystoscopy for patients on annual bladder cancer surveillance.

**List of Abbreviations**
CxM: Bladder Cx Monitor Test
NMIBC: Non‐Muscle Invasive Bladder Cancer
EAU: European Association of Urology
MDM: Multi‐Disciplinary team Meeting
RDGU: Research Development and Governance Unit
MIBC: Muscle Invasive Bladder Cancer
PUNLMP: Papillary Neoplasm of Low Malignant Potential
HG: High‐Grade
LG: Low‐Grade
UO: Ureteric orifice

## 1 BACKGROUND

Bladder cancer is associated with the highest lifetime treatment costs per patient of all cancers.^1^, ^2^ Surveillance schedules are not only burdensome for patients, but there can be associated anxiety and discomfort with the cystoscopies.^3^ Depending on patient factors, tumour characteristics and timing of tumour recurrences, the period of time between cystoscopies according to the European Association of Urology (EAU) ranges between 3 months to 12 months.^4^ New urinary biomarkers, however, provide the potential for more targeted surveillance regimens.^4^, ^5^

Bladder Cx Monitor (CxM) is a mRNA‐based urine test that assesses the levels of five mRNA biomarkers associated with bladder cancer (IGF, HOXA, MDK, CDC, IL8R) and correlates this with patient phenotypic data (whether the last tumour diagnosed was primary or recurrent disease) to create a risk score.^6^ A low‐risk CxM score means cystoscopy can be avoided, whilst conventional surveillance is recommended for high‐risk scores (cystoscopy with or without imaging).

CxM has proven high sensitivity (0.91–0.95) and NPV (0.96–0.97) in the surveillance setting of bladder cancer.^4^, ^6^, ^7^, ^8^ Notably, it has a higher sensitivity for higher‐risk tumours and an ability to detect upper tract lesions.^6^, ^7^, ^8^ CxM has been consistently shown to be superior to urine cytology in both NPV and sensitivity; urine cytology has not been shown to pick up any cases that CxM may miss.^6^, ^9^, ^10^ These characteristics give CxM the potential to be implemented into surveillance regimes to reduce costs and the burden to both patient and the healthcare system.^11^, ^12^

CxM has become part of surveillance guidelines, specifically used in place of cystoscopy in various health centres in NZ, and has successfully been used in place of cystoscopy in a multi‐centre study in the USA.^13^, ^14^ These studies led to our institution implementing a surveillance regimen of alternating CxM and cystoscopy for NMIBC patients reaching the thresh‐hold for annual bladder cancer surveillance; stretching the possible duration between cystoscopies to approximately two years. Patients receiving annual cystoscopies were specifically chosen, as this population of patients have a relatively lower risk of recurrence than those requiring more frequent cystoscopies. The purpose of this study is to audit the outcomes of patients placed on this new surveillance protocol.

## 2 METHODS

### 2.1 Study design

This was a retrospective audit of the outcomes and patterns of treatment for patients who were placed onto our new surveillance protocol during the year of 2022. Consenting patients who were eligible underwent CxM instead of their planned flexible cystoscopy.

Patients performed the CxM tests at our health service, but the specimens were processed in New Zealand. A nurse practitioner was responsible for arranging, reviewing and collating results. All positive results were discussed with the Urology consultants to ascertain whether upper tract imaging was necessary and whether rigid or flexible cystoscopy should be performed. If necessary a clinic consultation to discuss results was made.

**FIGURE 1:**
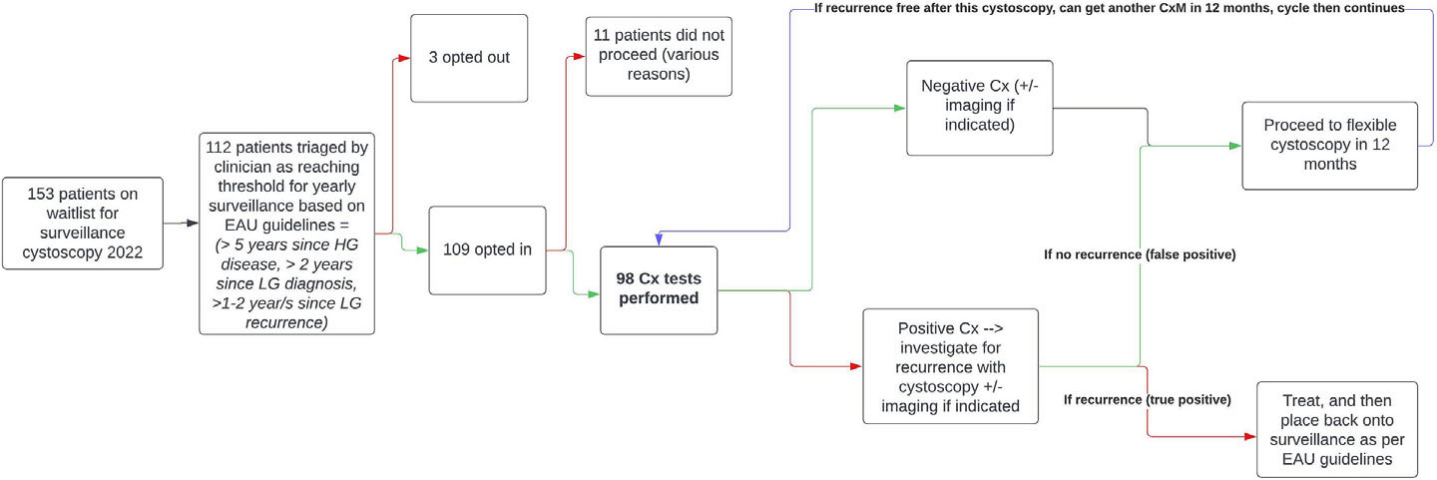
Patient selection and novel CxM surveillance protocol design.

A positive (high‐risk) CxM, meant the patient underwent a flexible or rigid cystoscopy, and if indicated up‐to‐date imaging (Figure 1). If a tumour was seen at cystoscopy (true positive) they were treated, and if appropriate placed back onto traditional surveillance. A negative cystoscopy/imaging (false positive CxM) would result in the patient being booked for a flexible cystoscopy in 12 months' time (Figure 1).

If a patient had a negative (low‐risk) CxM and up‐to‐date imaging (if indicated), a flexible cystoscopy +/− upper tract imaging (if indicated) was organised in 12 months' time (Figure 1).

### 2.2 Inclusion criteria

All patients due for a surveillance cystoscopy in 2022 (January 1st to December 31st) reaching the threshold of annual surveillance as per the EAU guidelines at the time of scheduled cystoscopy, and consenting to the new surveillance protocol, were included. Thus, patients with a history of only low‐grade disease were defined as being greater than 2 years since low‐grade diagnosis. Patients with a history of high‐grade disease were defined as being over 5 years (at the time of CxM) since their high‐grade recurrence/diagnosis. If any of these patients had a low‐grade recurrence, they were able to be included if they were greater than 1 year since recurrence. If the low‐grade recurrence was a large tumour (>3 cm) or multi‐focal disease, this time value was increased to two years. One caveat to these criteria was that as per clinician discretion, patients were still included in the CxM schedule if they were around 2–3 months under the aforementioned limits (e.g. if a patient was 4 years and 9 months post high‐grade primary diagnosis at the time of scheduled cystoscopy, they were still deemed as able to be included).

### 2.3 Exclusion criteria

Patient's having symptoms (such as haematuria or flank pain) were not to proceed with CxM instead of cystoscopy.

### 2.4 Outcomes

The main focus was to see if a clinically significant recurrence of bladder cancer was missed with the use of this new surveillance protocol; this would be the case if a patient progressed to invasive or metastatic disease after a negative CxM. Thus, the incidence and types of recurrences (if any) that patients had at the time of the next cystoscopy (+ upper tract imaging if indicated) after negative CxM were recorded. PPV of patients who had a positive CxM, was also assessed. To adequately measure the treatment patterns of these patients, false positive CxM patients were followed up until December 31st^,^ 2023 (at which time all false positive patients would have received their next cystoscopy +/− imaging), and negative CxM patients were followed up until the time of next cystoscopy +/− imaging, or until discharge from surveillance. Negative CxM patients who did not proceed with further surveillance had their medical records audited to assess if they presented to the hospital with symptoms of a recurrence. There was no extensive follow‐up of true positive CxM patients.

The secondary outcomes evaluated were patient satisfaction, wait‐list reduction and costs involved. Patient satisfaction was measured by whether a patient consented to continue with the surveillance protocol, and if not consenting, their rationale. Wait‐list reduction and costs involved were calculated by our hospital's audit team.

### 2.5 Statistical analysis

The recurrences seen in the true positive CxM population and the population with recurrences after negative CxM were assessed in detail; specifically diagnoses (including tumour grade), bladder cancer history and location within the urinary tract. The proportion of these recurrences was compared to patients of the positive CxM and negative CxM cohorts.

Patient demographics, age and recurrence‐free duration at the time of CxM was calculated with a mean and 1 standard deviation of range. Smoking status at diagnosis, gender, upper tract disease and original pathology were reported as actual number of patients and percentage of the total cohort that was followed up. For each patient, a mean 5‐year risk of progression to muscle‐invasive bladder cancer (MIBC) at the time of their last recurrence (or diagnosis if no recurrences) was also recorded as the percentage devised by the EAU prognostic calculator.^15^

Of the negative CxM patients that had a recurrence at the next cystoscopy/imaging, the specific times between the test and recurrences were recorded, as well as the mean and 1 standard deviation of range.

The demographics of the negative CxM patients who had no recurrence at the next cystoscopy +/− imaging and the ones who had a recurrence at the next cystoscopy +/− imaging were logged in exactly the same manner as the demographics of the total cohort. These two aforementioned groups then had the demographics of age, smoking status at diagnosis, recurrence‐free duration at the time of Cx and mean 5‐year progression to MIBC compared with a two‐tailed Mann–Whitney U test (p‐value significant below 0.05).

With respect to the reduction in the flexible cystoscopy waitlist (surveillance and overall), two calculations were done to get the reduction as a percentage. First, the total number of flexible cystoscopies able to be skipped was found. This was devised by finding the total number of patients who had CxM minus the total number of patients who had a positive CxM. This number was then divided by both the number of patients on the surveillance waitlist and the overall flexible cystoscopy waitlist and a percentage was calculated.

The cost saving (not accounting for the Australian medicare rebate and revenue generation) was calculated using the previously calculated number of flexible cystoscopies able to be skipped. This value was multiplied by the difference in cost between a flexible cystoscopy and cumulative costs of the tests and associated nurse‐led clinic costs.

### 2.6 Ethics

Given the study was undertaken to assess and audit the surveillance protocol which was a part of standard care, an ethics exemption was granted by our centre's Research Development and Governance Unit (RDGU). Patients involved were adequately consented regarding CxM and the new surveillance protocol. Only anonymised data from patient electronic medical records was extracted for analysis.

## 3 RESULTS

Of the 153 patients on the surveillance waitlist for flexible cystoscopy at the beginning of 2022, 112 were triaged to be eligible as per our inclusion criteria. A total of 109 of these patients opted in for Cx at the start of the calendar year, however, when 11 of these patients were due for their CxM they did not end up proceeding with CxM (Figure 1). The major reasons for this included patients declining further surveillance, death due to other causes and moving to another health service.

**FIGURE 2:**
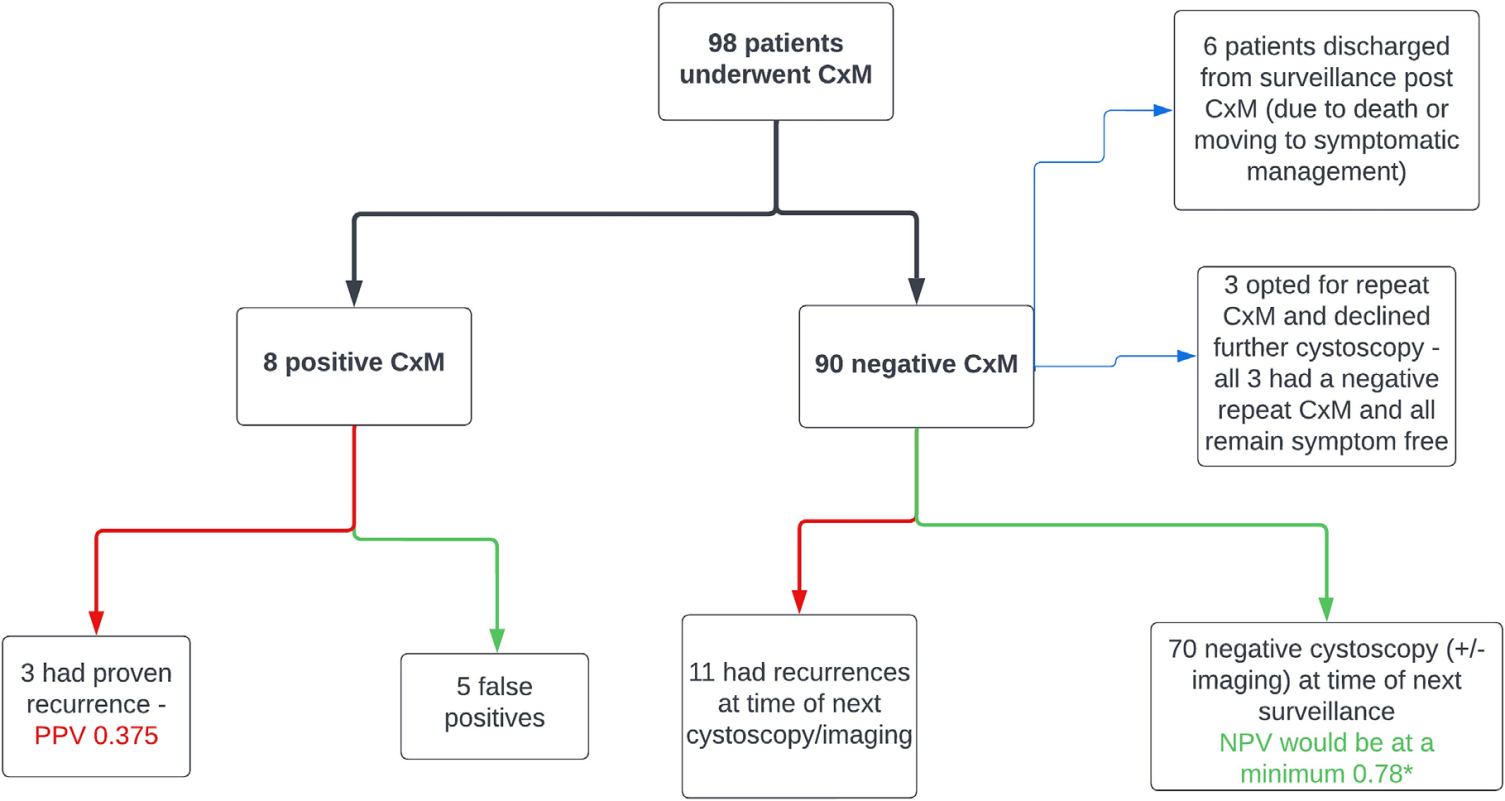
Result of CxM tests with number and outcomes of patients within each sub‐category.

In total 98 patients ended up proceeding with CxM tests – 90 of these were negative and 8 of these were positive (Figure 2). Of the eight positive CxM, only three were true positives (PPV 0.375). Of the 90 negative CxM tests, 70 had no recurrences when this cohort underwent subsequent cystoscopy (approximately 12 months later). Of the remaining 20 negative CxM patients, 11 had confirmed recurrences on subsequent cystoscopy, while 9 patients did not proceed with further cystoscopic surveillance. Six of these nine were discharged from surveillance due to death, or patient's transitioning to symptomatic management. The remaining three no longer wanted cystoscopy and requested ongoing CxM; these three patients continue to have negative CxM results on file review at the end of calendar year 2023. Finally, none of the nine who did not have cystoscopy (when file review was conducted at the end of the calendar year 2023) post‐CxM had presented to our institution with symptoms suggesting recurrence (haematuria/flank pain). Whilst a true NPV cannot be calculated due to the design of our study, it would be at least 0.78 based on the aforementioned results.

Demographics of the whole cohort of 98 patients that had a CxM test followed‐up revealed an older (mean age in years 74.1) and predominately male population, with a significant smoking history. The mean recurrence‐free time when CxM was performed was 5.0 years and there was overall minimal incidence of upper tract disease (Table 1). The majority of patients initial bladder tumours were low‐grade, however, 34% had original high‐grade pathology. There were five patients whose original bladder tumours were classified as other (Table 1); four were diagnosed with Papillary Neoplasm of Low Malignant Potential (PUNLMP) and the last patient in this category was being monitored in the context of an Inverted Papilloma. All 98 patients, except for one (Table 2), had a 5‐year percentage risk of progression to MIBC between 0.9% and 9.6% at the time of their last recurrence/diagnosis, hence it correlates that the mean 5 year percentage risk is 5.0%.

**TABLE 1:** Patient demographics of all patients that underwent CxM.

Age – in years (mean, 1 SD)	74.1 (+/− 10.7)
**Time between Cx and last recurrence/diagnosis** – in years (mean, 1 SD)	5.0 (+/− 3.6)
**Smoking status at first diagnosis** Active smoker (n, % of total) Ex‐smoker (n, % of total) Never smoker (n, % of total)	43 (44%)33 (34%)22 (22%)
**Gender** Female (n, % of total) Male (n, % of total)	19 (19%)79 (81%)
**Upper tract (UT) disease** First diagnosis in UT (n, % of total) Bladder 1° with UT recurrence (n, % of total)	7 (7%)1 (1%)
**Original pathology** LG pTa (n, %) HG pTa (n, %) CIS (n, %) HG pT1 (n, %) Other (n, %)	59 (60%)23 (23%) 4 (4%) 7 (7%) 5 (5%)
**EAU 5‐year percentage risk of progression to MIBC at time of last recurrence/diagnosis** (mean, 1 SD)	5.0% (+/− 4.7%)

**TABLE 2:** Details of background and pattern of treatment for patients who had recurrences in study population.

Background of patient	CxM result	How many recurrences prior to CxM	Recurrence free time at time of CxM (year/s)	5‐year risk of progression to MIBC at time of diagnosis/last recurrence	Prior upper tract disease?	Pattern of treatment
76 M, never smoker, first diagnosis LG pTa in bladder and involving LEFT ureteric orifice (UO)	Positive	0	3.0	4.9%	No	1 cm HG pTa found 4 cm from LEFT ureteric orifice, proceeded to nephroureterectomy following MDM discussion (uncomplicated surgery)
90 M, active smoker, first diagnosis distal ureteric LG pTa (treated with distal ureterectomy with re‐implant)	Positive	3	1.0	4.9%	Yes	Small focal LG pTa recurrence in bladder biopsied and diathermised
80 M, active smoker, first diagnosis HG pT1 bladder, induction BCG but no maintenance (diagnosed with lung cancer so could not proceed), had a recurrence involving RIGHT UO	Positive	4	4.8	40%	No	Normal cystoscopy at time of positive CxM. Upper tract imaging performed after cystoscopy showed metastatic upper tract TCC (RIGHT renal pelvis primary). Referred to oncology for consideration palliative therapies.
77 M, active smoker, first diagnosis HG pTa bladder, induction and maintenance BCG	Negative	2	6.9	4.9%	No	Small presumed LG pTa treated and diagnosed after first routine cystoscopy 18 months post CxM (<5 mm lesion diathermised only)
82 M, active smoker, first diagnosis LG pTa bladder	Negative	0	3.1	0.9%	No	LG pTa recurrence around and invading into distal 2 cm of LEFT UO treated endoscopically 11 months post CxM, having been diagnosed after first routine cystoscopy post CxM.
71 M, ex‐smoker, first diagnosis LG pTa bladder	Negative	0	0.8	0.9%	No	Only cystoscopy post CxM test brought forward from routine booking given ongoing haematuria and radiological evidence of recurrence. Diagnosis of recurrence 5 months post CxM. This revealed multi‐focal LG pTa (multiple <2 cm LG tumours, but in all 4 quadrants). N.B. Inappropriately placed onto CxM surveillance schedule due to error; patient was 9 months post LG diagnosis and also had active haematuria at time of CxM test.
86 M, ex‐smoker, first diagnosis LG pTa bladder	Negative	2	2.9	4.9%	No	Small LG pTa biopsied and diathermised 10 months post CxM, having been diagnosed after first routine cystoscopy.
80 M, ex‐smoker, first diagnosis LG pTa bladder	Negative	0	3.8	4.9%	No	Small LG pTa biopsied and diathermised 10 months post CxM, having been diagnosed after first routine cystoscopy post CXM.
88 M, never smoker, first diagnosis CIS bladder, no adjuvant BCG	Negative	1	3.0	4.9%	No	Small red patch biopsied and diathermised 14 months post CxM, found to be HG pTa. Diagnosed after first routine cystoscopy post CxM.
75 M, active smoker, first diagnosis LG pTa bladder	Negative	0	6.3	0.9%	No	Small lesion diathermised (presumed LG pTa) 12 months post CxM, having been diagnosed after first routine cystoscopy post CXM.
58 M, active smoker, first diagnosis LG pTa bladder	Negative	0	2.1	0.9%	No	2 small LG pTa lesions resected and diathermised either side of RIGHT ureteric orifice 13 months post CxM. Diagnosed after first routine cystoscopy post CxM.
78 M, ex‐smoker, first diagnosis LG pTa bladder	Negative	0	3.7	0.9%	No	Small LG pTa biopsied and diathermised 11 months post CxM, having been diagnosed after first routine cystoscopy post CxM.
84F, ex‐smoker, first diagnosis LG pTa bladder	Negative	2	3.0	9.6%	No	One 1 cm LG pTa and 2 < 5 mm LG pTa satellite lesions resected and diathermised 12 months post CxM. Diagnosed after first routine cystoscopy post CxM.
58 M, active smoker, first diagnosis HG pTa bladder and involving LEFT ureteric orifice, no BCG	Negative	1	4.1	4.9%	No	LG pTa 2 cm distal LEFT ureteric lesion cleared endoscopically 15 months post CxM. Diagnosed after first routine cystoscopy post CxM.

When specifically looking at the positive CxM patients, 2 of the 3 true positive's had upper tract recurrences, and both of these were in patients with no prior upper tract disease (Table 2). All of the false positive CxM patients remained recurrence‐free at the end of their follow‐up period (end of calendar year 2023).

**FIGURE 3:**
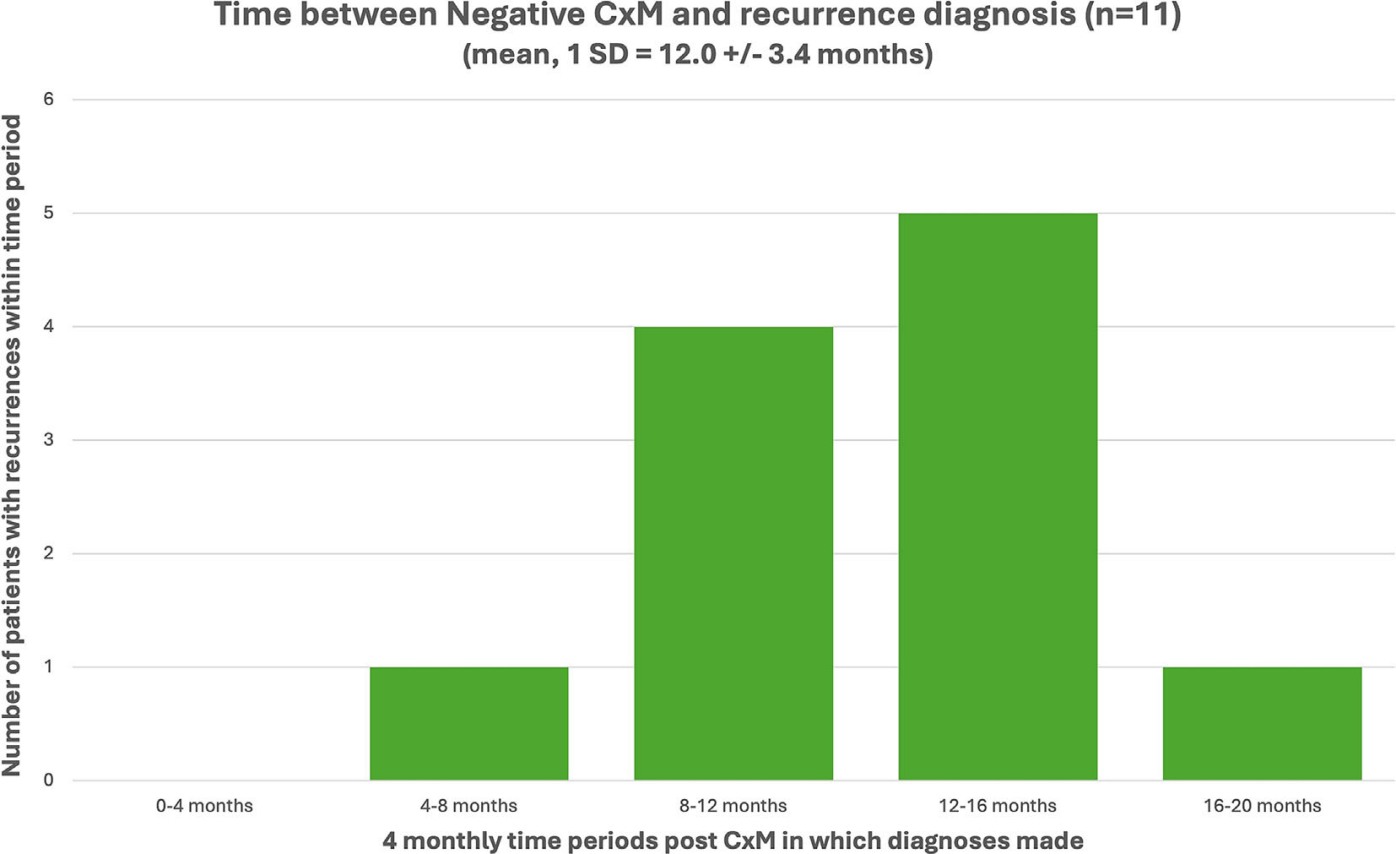
For the 11 patients who had recurrences post a negative CxM ‐ graph detailing the timing at which recurrences were diagnosed over 4‐month periods.

None of the 11 recurrences, detected after a negative CxM were invasive nor of high volume, and no patients progressed to have metastatic disease (Table 2). The grading of these recurrences was one High‐Grade (HG) pTa, eight Low‐Grade (LG) pTa and two presumed LG pTa (Table 2). One of the recurrences, a 71‐year‐old male patient (Table 2), had been found to have been inappropriately triaged onto the CxM surveillance protocol. This patient was only 9 months post LG diagnosis at the time of CxM and it was discovered he had active haematuria at the time of his CxM. This resulted in his cystoscopy being performed earlier than planned, due to ongoing symptoms (Table 2, Figure 3). All the other recurrences were diagnosed at their first routine cystoscopy post negative CxM, which as per the protocol were booked at a 12‐month time interval. The exact timing of these routine cystoscopies varied for each patient (from 10 months to 18 months post CxM) and these are detailed in Table 2 and Figure 3.

The demographics of the Negative CxM patients (Table 3), revealed the recurrence group tended to have had shorter recurrence‐free time when CxM was conducted compared to the no recurrence group. Otherwise, there were no statistically significant differences between the groups.

**TABLE 3:** Demographics of the patients who had recurrence versus no recurrence at the time of next cystoscopy/imaging post a negative CxM.

	No recurrence (n = 70)	Recurrence (n = 11)
**Mean Age (years)** ‐ p value 0.49	73.2	75.7
**Smoking status at first diagnosis (%) ‐** p value 0.27 **Active/Ex‐smoker** **Never smoker**	50 (71%)20 (29%)	10 (91%) 1 (9%)
**Original histopathology (%)** **LG pTa** **HG pTa** **CIS/pT1** **Other**	41 (59%)16 (23%)8 (11%) 5 (7%)	8 (73%)2 (18%)1 (9%)0 (0%)
**Mean time between Cx and last recurrence/diagnosis (years)** ‐ p value 0.045	5.27	3.59
**Mean 5‐year percentage risk of progression to MIBC at time of last recurrence/diagnosis**‐ p value 0.087	4.8%	3.2%

In regards to patient satisfaction, all suitable patients were happy to continue with the protocol and consented to remain on the regime, with no significant concerns voiced. As stated earlier, three patients opted for CxM only over cystoscopy as they no longer wanted an invasive investigation.

There was a significant impact on the surveillance cystoscopy waitlist with a reduction of 59%. The overall flexible cystoscopy waitlist was reduced from 654 to 564, correlating to a reduction of 14%, which helped offset the influx of cases seen post the COVID‐19 pandemic. CxM was calculated to be $850 (Australian dollar) cheaper than a flexible cystoscopy, not accounting for revenue generation and the government medicare rebate, correlating to $78 200 saved. Most importantly, there was no loss of revenue from flexible cystoscopies as the vacant slots created by CxM were filled by other patients on the waiting list for a cystoscopy.

## 4 DISCUSSION

This study demonstrates that in real‐world practice CxM can be used to safely increase the duration between cystoscopies for patients eligible for annual surveillance cystoscopy, reducing a hospital's waitlist without compromising revenue.

As mentioned previously, we were unable to measure a true NPV as there was no cystoscopy performed for patients at the same time as their CxM test; this was the intent of the study, given the previous studies on NPV.^4^, ^6^, ^7^, ^8^ However, given the follow‐up results and treatment patterns of the cohort of negative CxM patients, it is unlikely that any clinically significant bladder cancer was missed when CxM was performed instead of cystoscopy; this is evident from the recurrence invasiveness, recurrence size and quantity and timing of recurrence after negative CxM for patients as detailed in Table 2. The fact that patients did not progress to muscle‐invasive nor metastatic disease at the time intervals cystoscopies were performed after negative CxM (Table 2, Figure 3), also supports this notion. We note that this is debatable for the 71‐year‐old male who inappropriately underwent CxM instead of cystoscopy (Table 2). It is possible this patient had a small low‐grade tumour that CxM did not pick up on. Regardless, this case attests to the rationale of the design of the CxM surveillance protocol.

NMIBC has a 5‐year risk of recurrence between 31% and 78%, with the majority of these within the first two years.^15^, ^16^ Whilst low‐risk low‐grade disease patients need not be monitored when tumour‐free for 5 years, patients with a history of high‐grade disease that are tumour‐free for over 5 years are estimated to have a 5–10 year recurrence risk between 12.5% and 20%.^17^, ^18^ Thus, the rates of recurrence in the negative CxM (14.5%) patient cohort was within the expected range.

Accordingly, our surveillance protocol has been formulated to alternate CxM with cystoscopy when the time between examinations can be extended to its maximum. When the NPV of CxM (0.96–0.97) is correlated with a lower risk of recurrence, it confers a lower likelihood of missed recurrences. Additionally, if a recurrence is missed by CxM, it is more likely that this would be a low‐grade tumour, given the test was created with a higher sensitivity for high‐grade tumours.^6^, ^9^, ^10^ Overall, the variance between CxM and cystoscopy in ruling out recurrences has been significantly minimised in the way it is implemented in our protocol, as we specifically conducted the test on patients on annual surveillance.

There were 23 patients (out of 112 originally) who were not able to proceed with CxM or be adequately followed up for the purposes of our study (Figures 1 and 2). This reduction of patients (21%) was not unexpected and reflects the dynamic requirements of bladder cancer patients who often face multiple health issues or other personal concerns. The same rationale also provides an explanation for the variability in the timing of routine cystoscopy after a negative CxM test for those patients who remained on surveillance, which was a limitation that was difficult to control in this study. Health systems can benefit by potentially reducing surveillance of the older, co‐morbid patients that on average have a high recurrence‐free time (5.0 years +/− 3.6 in this study), with the cheaper and safer option of CxM.

The low PPV of 0.375 in this study was in keeping with the PPV of CxM in previous published studies.^7^, ^9^, ^13^, ^14^ The PPV value in our study is quite limited due to the low incidence of positive CxM tests in our patient cohort. However, we believe that the PPV value is not important in the context of our study; the important outcome measure was whether there was progression to muscle‐invasive or metastatic disease with the use of our surveillance protocol. In our experience, it is difficult to endorse recommending upper tract imaging for all positive CxM results. We did have had two positive CxM patients with a negative cystoscopy that had no prior upper tract disease but were found to have upper tract recurrences. Both of these patients actually did have disease involving their ureteric orifice (UO) previously, one of which had a 40% 5‐year risk of progression to muscle‐invasive disease at the time of last recurrence (Table 2). Based on the results of this study, we recommend performing upper tract imaging as usual but with a lower thresh‐hold for patients who had previous disease involving one or both ureteric orifice's (UO's), or those who are in the high/very high‐risk category of progression to MIBC (Table 2).

Even in spite of false positives requiring otherwise routine investigation, we were still able to achieve significant reductions in our hospital surveillance and overall flexible cystoscopy waitlist. Due to back‐log of surgeries created by COVID‐19, our centre's flexible cystoscopy waitlist had doubled at the start of 2022. The ability to defer surveillance cystoscopy created vacancies allowing other patients to be brought forward to have their cystoscopy.

There were minimal statistically significant differences between those who had a negative CxM and no recurrence post, to those who had a recurrence after the test. The higher incidence of low‐grade disease in the group of patients who had recurrence after negative CxM compared to those who did not, likely contributes to the fact there is a statistically significant lower mean recurrence‐free time in the former group (Table 3).

We acknowledge that our study is limited as we did not have long‐term follow‐up of all our patients, but believe our study is still of merit because we did follow up with all patients still under surveillance until their next cystoscopy/imaging after their CxM test. Our study is also limited by the overall quantity of patients in the study population, greater numbers would provide more accurate deductions. A study on the XPert Bladder Cancer Monitor, a different urine test to CxM, showed that when it was longitudinally repeated could significantly reduce the number of cystoscopies.^19^ With a longer follow‐up period and greater patient numbers, we could similarly assess the potential ability of repeated negative CxM tests to further reduce the amount of cystoscopies performed; this could be used as a guide for selecting patients amenable to transition from alternate cystoscopy and CxM testing to repeated CxM testing. This would be particularly pertinent for patients with a history of high‐grade disease, or intermediate‐risk bladder cancer, which the EAU recommends surveilling up until 10 years.^4^

Overall, the significance of this study is that it represents the largest study detailing the real‐world use of CxM for NMIBC patients with a history of both high‐grade and low‐grade disease. Other strengths include the insight into the implications of decision‐making based on CxM results, and the detailed follow‐up of patients.

## 5 CONCLUSION

An alternating CxM and Flexible Cystoscopy surveillance protocol can be safely used for NMIBC patients eligible for annual surveillance, without clinically significant recurrences being missed. This can also alleviate a health centre's surveillance cystoscopy wait list and allow improved patient access to cystoscopy. CxM was found to be cheaper and the patient enthusiastically accepted it as an alternative to cystoscopy.

## AUTHOR CONTRIBUTIONS

**Arjun Guduguntla:** Data curation; investigation; formal analysis; writing—original draft; writing—review and editing; project administration. **Lauren Chandler:** Investigation; writing—review and editing. **Thomas Whish‐Wilson:** Investigation; writing—review and editing. **Dennis Gyomber:** Conceptualisation; methodology; resources; supervision; writing—review and editing.

## CONFLICT OF INTEREST STATEMENT

The authors have no relevant financial or non‐financial interests to disclose.
